# Entropy-Based Shear Stress Distribution in Open Channel for All Types of Flow Using Experimental Data

**DOI:** 10.3390/e23111540

**Published:** 2021-11-19

**Authors:** Yeon-Moon Choo, Hae-Seong Jeon, Jong-Cheol Seo

**Affiliations:** Department of Civil and Environmental Engineering, Pusan National University, Busan 46241, Korea; chooyean@naver.com (Y.-M.C.); man5020@naver.com (H.-S.J.)

**Keywords:** entropy, shear stress distribution, Shannon’s theory, Korean river design standards

## Abstract

Korean river design standards set general design standards for rivers and river-related projects in Korea, which systematize the technologies and methods involved in river-related projects. This includes measurement methods for parts necessary for river design, but does not include information on shear stress. Shear stress is one of the factors necessary for river design and operation. Shear stress is one of the most important hydraulic factors used in the fields of water, especially for artificial channel design. Shear stress is calculated from the frictional force caused by viscosity and fluctuating fluid velocity. Current methods are based on past calculations, but factors such as boundary shear stress or energy gradient are difficult to actually measure or estimate. The point velocity throughout the entire cross-section is needed to calculate the velocity gradient. In other words, the current Korean river design standards use tractive force and critical tractive force instead of shear stress because it is more difficult to calculate the shear stress in the current method. However, it is difficult to calculate the exact value due to the limitations of the formula to obtain the river factor called the tractive force. In addition, tractive force has limitations that use an empirically identified base value for use in practice. This paper focuses on the modeling of shear-stress distribution in open channel turbulent flow using entropy theory. In addition, this study suggests a shear stress distribution formula, which can easily be used in practice after calculating the river-specific factor T. The tractive force and critical tractive force in the Korean river design standards should be modified by the shear stress obtained by the proposed shear stress distribution method. The present study therefore focuses on the modeling of shear stress distribution in an open channel turbulent flow using entropy theory. The shear stress distribution model is tested using a wide range of forty-two experimental runs collected from the literature. Then, an error analysis is performed to further evaluate the accuracy of the proposed model. The results reveal a correlation coefficient of approximately 0.95–0.99, indicating that the proposed method can estimate shear-stress distribution accurately. Based on this, the results of the distribution of shear stress after calculating the river-specific factors show a correlation coefficient of about 0.86 to 0.98, which suggests that the equation can be applied in practice.

## 1. Introduction

Understanding fluid interaction is very important in almost all studies of open channel flows. Shear stress is used and applied in hydraulics, hydrology, fluid mechanics, and in various fields, and is one of the most important mechanical factors [1]. It is a great challenge to the river engineers and researchers working in the field to estimate the distribution of bed shear stress in open channel flows [2].

Leighly [3] proposed that the bed shear stress can be balanced by the downstream component of the weight of water contained within the bounding orthogonal. Lundgren and Jonsson [4] modified the logarithmic law to a parabolic cross-sectional open channel and suggested a method to estimate the velocity and shear stress distribution. Chiu et al. [5,6] studied the complex interaction between primary and secondary flows, shear stress distribution, channel characteristics such as roughness, slope and geometry, and other related factors in open channel flows. However, the velocity profile was required to estimate boundary shear stress.

Keulegan [7] and Johnson [8] contributed to the early development of shear stress, and Einstein’s [9] hydraulic radius separation method is still used in various studies. Following this idea, Knight and his associates [10,11,12,13,14,15,16] proposed several empirical relations that are very helpful in understanding open channel flows and sediment transport. Noutsopoulos and Hadjipanos [17], Hu [18], and Patel [19] have led to an improved understanding of the lateral distributions of wall shear stress in rectangular channels, prismatic channels, and ducts. Past literature shows that the shear stress profile in open channel flows has been studied either experimentally or theoretically using deterministic approaches.

For these reasons, this study focuses on theoretical and statistic methods, which are probability and entropy concepts. Combining these, a useful method can be developed to explain the shear stress profile in open channel flows.

A probabilistic method of shear stress distribution in open channel flows using entropy concepts has been studied. Bonakdari et al. [20] compared Shannon and Tsallis entropies for shear stress distribution prediction in open channels. Sheikh and Wan [21] used the new Tsallis-based equation to predict shear stress distribution in circular and trapezoidal channels. Then, Mirauda and Maria [22] used an entropic parameter for modeling bed shear stress distribution in rectangular channels.

For the past few decades, entropy theory has been applied in the field of river hydraulic geometry and fluvial hydraulics. The entropy concept was introduced in hydraulics by Chiu [23] with Shannon’s entropy. Chiu studied the two-dimensional velocity distribution in an open channel [24,25]. Later, Choo [26] used Chiu’s velocity equation to calculate the momentum and energy coefficients. Especially, Chiu et al. [27] modified the entropy concept to be applied in pipe flows. Here, Chiu et al. compared with the Schlichting equation and the relationship between the frictional loss coefficient and entropy coefficient M, but there was no study on the shear stress. Singh [28] studied a wide range of hydrology and water resources based on entropy theory.

Since then, various entropy-related research has been carried out. Chiu et al. [29,30] applied the maximum velocity and regularity and a one-dimensional velocity distribution in an open channel with the entropy concept. Singh and Luo [31] examined the one-dimensional velocity distribution in an open channel with entropy theory, where they used Shannon’s entropy to derive the power law and logarithmic velocity distribution. Cui and Singh [32,33,34] studied velocity distribution and sediment concentration in open channels using Tsallis entropy. Singh and Cui [35] developed sediment concentration in a debris flow by Tsallis entropy.

Shear stress has to be estimated to determine flow characteristics. Considering the importance in an open channel, the complete evaluation is highly difficult due to the complexity of the cross-section and the various hydraulic parameters. The research related to the shear stress is based on empirical outcomes. That being so, it is difficult to apply the equations generally. Therefore, the current Korean river design standards use simple and obtainable tractive force and critical tractive force, which can be obtained through empirical methods instead of shear stress.

The objective of this study, therefore, is to model shear stress distribution using entropy theory, verify the model using twenty-one experimental datasets obtained from Song’s experimental data [36], and to prove the utility of the proposed equation. Based on the proven equation, it is then suggested that the tractive force and critical tractive force in the Korean river design standards be revised to the shear stress obtained using the proposed shear stress distribution formula by presenting a method that can be easily used in the actual conditions.

## 2. Methodology

### 2.1. Entropy Theory

The variable x related to information I(x) is shown as Equation (1), which provides the amount of information. Here, information I(x) is the measurement of uncertainty related to a certain state as:

(1) I(x)=lnp(x)

Considering every state, the average value of information I(x) can be expressed as Equation (2). Function H(x) is defined using Shannon’s [37] entropy as:

(2) $$H\left(x\right)=-\int_{-\infty }^{+\infty }p\left(x\right)I\left(x\right)dx=-\int_{-\infty }^{+\infty }p\left(x\right)lnp\left(x\right)dx$$

where p(x) and lnp(x) are dimensionless, but dx has a dimension; thus, H(x) has the same dimension as dx. In Equation (2), a probability density function (PDF) p(x) of a continuous variation state means maximizing the entropy of uncertainty x.

### 2.2. Constraint Conditions

The probability distribution of maximizing the entropy produces more information from already acquired basic knowledge. To solve the PDF, i.e., the available information of variable x in Equation (3), the constraint conditions, such as average, variance, distortion, etc., are applied as:

(3) $$\int_{a}^{b}\Phi_{i}\left(x,p\right)dx\;\;\;i=1,\;2,\;3,\;\ldots n$$

Therefore, the PDF p(x), which maximizes the entropy, can be obtained using the method from Lagrange in Equation (4); we have:

(4) $$\frac{\partial I\left(x,p\right)}{\partial p}+\overset{n}{\underset{i=1}{\sum }}\lambda_{i}\frac{\partial \phi_{i}\left(x,p\right)}{\partial p}=0$$

where $\lambda_{i}$ denotes the Lagrange multipliers.

### 2.3. Entropy Maximization

The entropy concept can be applied to the shear stress using Equation (5) from Shannon’s entropy as:

(5) $$H\left(\tau \right)=-\int_{0}^{\tau_{0}}p\left(\tau \right)I\left(\tau \right)d\tau =-\int_{0}^{\tau_{0}}p\left(\tau \right)lnp\left(\tau \right)d\tau$$

where τ is point shear stress.

The available information for τ uses constraint conditions. First, the total probability must be satisfied for the PDF p(τ) as:

(6) $$\int_{0}^{\tau_{0}}p\left(\tau \right)d\tau =1$$

which follows from the total probability rule.

Then, average information can be expressed as:

(7) $$\int_{0}^{\tau_{0}}\tau \cdot p\left(\tau \right)d\tau =\bar{\tau }$$

### 2.4. Lagrange Method

Arranging the independent constraint conditions can be given as:

(8) $$\int_{a}^{b}\Phi_{i}\left(\tau ,p\right)d\tau \;\;\;i=1,\;2$$

Therefore, PDF p(τ), which maximizes the entropy, can be obtained using the method of Lagrange as:

(9) $$\frac{\partial I\left(\tau ,p\right)}{\partial p}+\overset{2}{\underset{i=1}{\sum }}\lambda_{i}\frac{\partial \phi_{i}\left(\tau ,p\right)}{\partial p}=0$$

(10) I(τ,p)=p(τ)lnp(τ)

where $\phi_{1}\left(\tau ,p\right)=p\left(\tau \right),\;\phi_{2}\left(\tau ,p\right)=\tau \cdot p\left(\tau \right)$.

(11) $$\frac{\partial \phi_{1}\left(\tau ,p\right)}{\partial p}=1,\;\frac{\partial \phi_{2}\left(\tau ,p\right)}{\partial p}=\tau$$

Substituting Equation (9) into Equations (10) and (11) can be constructed as follows:

(12) $$-1-lnp\left(\tau \right)+\lambda_{1}+\lambda_{2}\tau =0$$

where $\lambda_{1}-1=a_{1},\;\lambda_{2}=a_{2}$ are the Lagrange multipliers and differentiating Equation (12) with respect to p(τ) results in the shear stress PDF as:

(13) $$p\left(\tau \right)=exp^{a_{1}+a_{2}\tau }$$

**Figure 1:**
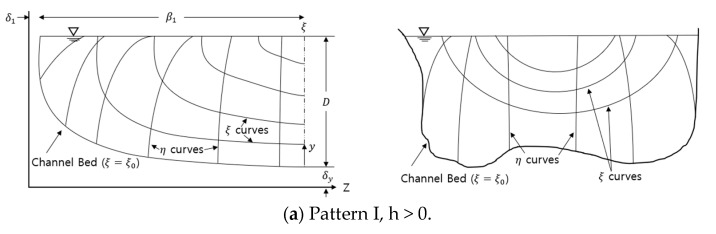

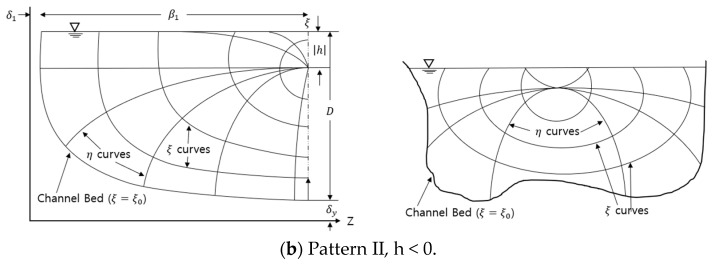
ξ-η coordinates in an open channel flow (Chiu [25,38]).

We apply the cumulative probability function to fluid flow using Equation (14), and Equation (13) will become Equation (14) by applying the PDF as:

(14) $$F\left(\tau \right)=\int_{0}^{\tau }p\left(\tau \right)d\tau =\int_{0}^{\tau }e^{a_{1}+a_{2}\tau }d\tau =1-\left[\frac{\xi -\xi_{0}}{\xi_{max}-\xi_{0}}\right]$$

where ξ denotes the spatial coordinates (0≤ξ≤1), τ is the point shear stress at ξ, $\xi_{0}$ is the minimum value of ξ (occurring at the channel boundary where τ=0), and $\xi_{max}$ is the maximum value of ξ (where τ is at its maximum) (see Figure 1).

The ξ-η coordinates are the isovel system, which was first developed by Chiu [25,38] to explain two-dimensional velocity distribution in the cross-section of an open channel.

### 2.5. Proposed Shear Stress Distribution Model

Solving Equation (14) can be written as:

(15) $$\tau =\frac{1}{a_{2}}ln\left[1+\frac{a_{2}}{e^{a_{1}}}\left(1-\left[\frac{\xi -\xi_{0}}{\xi_{max}-\xi_{0}}\right]\right)\right]$$

Open channel flow also uses the constraint conditions of Equations (6) and (7). Substituting Equation (13) into Equation (6) is given as:

(16) $$\frac{a_{2}}{e^{a_{1}}}=\left(e^{a_{2}\tau_{0}}-1\right)\Rightarrow e^{a_{1}}=\frac{T}{\left(e^{T}-1\right)\tau_{0}}$$

where $T=a_{2}\tau_{0}$ (normally called the entropy coefficient) to represent the model in a simple-to-use form and substituting Equation (13) into Equation (7) can be expressed as:

(17) $$\bar{\tau }=\int_{0}^{\tau_{0}}\tau \cdot p\left(\tau \right)d\tau =\int_{0}^{\tau_{0}}\tau e^{a_{1}+a_{2}\tau }d\tau \;\bar{\tau }=\left[\frac{e^{T}}{e^{T}-1}-\frac{1}{T}\right]\tau_{0}=\phi \left(T\right)\tau_{0}$$

where $\tau_{0}$ is the boundary shear stress, T is the entropy coefficient, and ϕ(T) is a function of T. Equation (17) is one of the proposed shear stress distribution equations for open channel flows. Substituting Equation (17) into Equation (16) and rearranging it in respect of the $e^{a_{1}}$ term gives:

(18) $$e^{a_{1}}=\frac{T}{\left(e^{T}-1\right)\tau_{0}}=\frac{T\cdot \phi \left(T\right)}{\left(e^{T}-1\right)\bar{\tau }}$$

Substituting Equation (18) into Equation (15) and rearranging it results in another proposed mean shear stress distribution formula as:

(19) $$\tau =\frac{\bar{\tau }\left(e^{T}-1\right)}{\left(Te^{T}-e^{T}+1\right)}ln\left[1+\left(e^{T}-1\right)\left(1-\left[\frac{\xi -\xi_{0}}{\xi_{max}-\xi_{0}}\right]\right)\right]$$

By inserting Equation (16) into Equation (15) we have:

(20) $$\tau =\frac{\tau_{0}}{T}ln\left[1+\left(e^{T}-1\right)\left(1-\left[\frac{\xi -\xi_{0}}{\xi_{max}-\xi_{0}}\right]\right)\right]$$

Equation (20) is the last proposed model for boundary shear stress distribution. Generally, $\xi_{0}$ is close to 0, $\xi_{max}$ is 1, and ξ for Equations (19) and (20) is the same, which was formulated as follows:

(21) $$\xi =\frac{y}{D-h}exp\left(1-\frac{y}{D-h}\right)$$

where D is the maximum depth, h is the depth where shear stress is 0 from the water surface (maximum velocity also occurs at this location), and y is the vertical depth from the bed for a given shear stress.

### 2.6. Shear Stress in Fluid

Normally when laminar and turbulent flows coincide, they can be written as:

(22) $$\tau =\rho \nu \frac{du}{dy}-\rho \bar{u^{\prime }v^{\prime }}=\rho \left(\nu +\varepsilon \right)\frac{du}{dy}$$

where ρ is the fluid density, ν is the fluid viscosity, du/dy is the velocity gradient, $\bar{u^{\prime }v^{\prime }}$ is the Reynolds stress, and ε is the eddy viscosity. Reynolds stress is the shear stress caused by turbulent fluctuating velocity. The kinematic coefficient of viscosity, ν, is caused by the molecular motion of fluid, and $\varepsilon_{y}$ is caused by fluid particle mixing, which is much larger than molecular motion.

Boundary shear stress from Equation (22) can be expressed as follows: when there is shear stress at the bed, y=0, the velocity is 0, and u=0. This is given by:

(23) $$\tau_{0}=\rho \left(\nu +\varepsilon \right){\left[\frac{du}{dy}\right]}_{y=0}=\rho gR_{h}S_{f}$$

where g is the gravitational acceleration, $R_{h}$ is the shape form of the cross-section, and $S_{f}$ is the energy gradient.

### 2.7. Tractive Force Formula in Fluid

Tractive force means the running water force when the silt on the river bed is moved by water. The commonly used tractive force ($\tau_{0}$) formula is:

(24) $$\tau_{0}=\omega Ri$$

where ω is the unit weight of water, i is the bed slope, and R is the hydraulic radius.

### 2.8. Critical Tractive Force Formula in Fluid

Critical tractive force means the tractive force at the beginning of the movement of the river bed silt due to the fact that the running water force is greater than the resistance of the river bed. The commonly used critical tractive force formula ($F_{s}$) is:

(25) $$F_{s}=\frac{\tau_{0}}{\left(\rho_{s}-\rho \right)gd}=\frac{u^{2}{}_{{*}_{c}}}{\frac{1}{\rho }\left(\rho_{s}-\rho \right)gd}$$

where $u_{{*}_{c}}^{}$ is critical friction velocity, $\rho_{s}$ is the density of silt particles, ρ is the density of water, and g is gravitational acceleration.

In terms of simplicity and convenience, Equations (22) and (23) have an advantage, but their accuracy is suspect. The reason for this is that the energy gradient is actually a difficult factor to estimate. As can be seen in Equation (22), the measured point velocity of the whole cross-section is required for shear stress to reach each gradient. In other words, the velocity gradient (du/dy), eddy viscosity coefficient (ε), and the energy gradient ($S_{f}$) in Equations (22) and (23) are factors that are very difficult to estimate. In addition, as shown by the Equation (24), it is difficult to accurately calculate the tractive force used by the Korean river design standards due to the hard-to-find river factors such as the river bed. Critical tractive force is readily calculated, but is not certain in terms of accuracy because the formulas are empirical formulas obtained from experiments. Therefore, this paper suggests an equation that can express the shear stress distribution and boundary shear stress in open channel turbulent flow using entropy-based modeling. This study demonstrates the utility of the proposed equation by using the Song data. It also proposes to revise the tractive force and critical tractive force of the Korean river design standards to shear stress by presenting measures easily applicable in practice.

## 3. Experimental Data

The proposed model of shear stress distribution was validated with experimental observations available in the literature. To test the validity of the model, i.e., Equations (19) and (20) with a wide range of slope, discharge, and sediment flow conditions, experimental data from Song [36] were selected (see Table 1). Forty-six flows were used in this study: twenty-one uniform flows, twenty-one non-uniform flows, and four unsteady flows according to four slope conditions. Out of twenty-one uniform flows, six runs were experimented under sediment conditions. For non-uniform flow, twelve accelerating flows and nine decelerating flows were tested. Four unsteady flows were tested according to four slope conditions. This study considered a various range of experimental runs for verification of the shear stress distribution.

## 4. Parameter Estimation and Comparison with Experimental Data

### 4.1. Parameter Estimation

**Figure 2:**
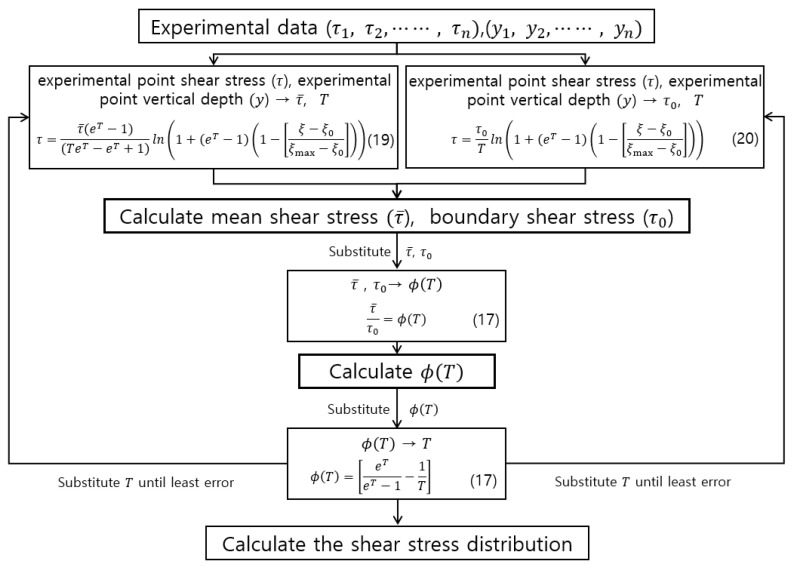
Parameter estimation flowchart.

Proposed shear stress distribution was used to estimate the entropy parameter T. First, we estimate parameter T by inserting experimental point shear stress ($\tau_{1},\;\tau_{2},\;\cdots \cdots ,\;\tau_{n})$ and point vertical depth from the bed for a given shear stress ($y_{1},\;y_{2},\;\cdots \cdots ,\;y_{n})$ into Equations (19) and (20). We estimate the best boundary shear stress $\tau_{0}$ and mean shear stress $\bar{\tau }$ value, which has the least error for each run. We then use $\tau_{0}$ and $\bar{\tau }$ from Equations (19) and (20) to estimate ϕ(T) from Equation (17). Lastly, we calculate the shear stress distribution for given vertical depths ($y_{1},\;y_{2},\;\cdots \cdots ,\;y_{n})$. The parameter estimation process is shown in Figure 2.

### 4.2. Comparison with Experimental Data

**Figure 3:**
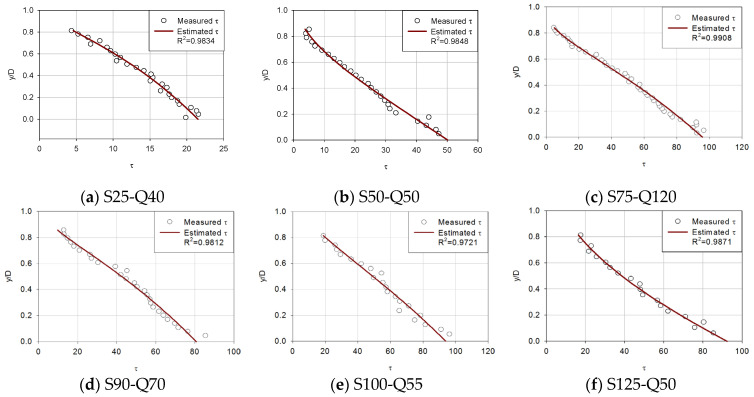
Verification of the proposed shear stress distribution model with six uniform flows.

**Figure 4:**
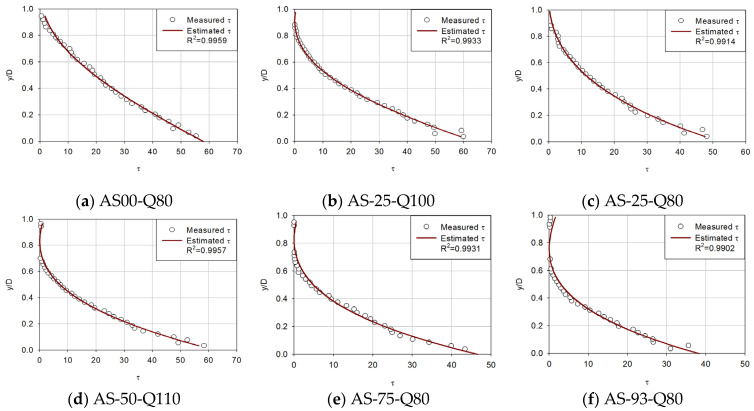
Verification of the proposed shear-stress distribution model with six accelerating non-uniform flows.

**Figure 5:**
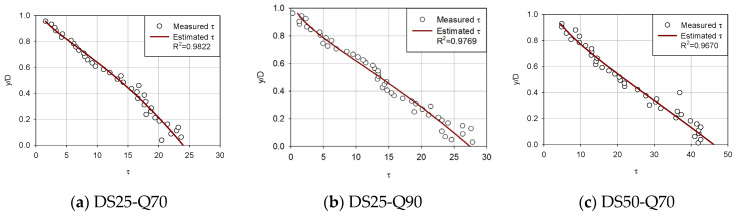

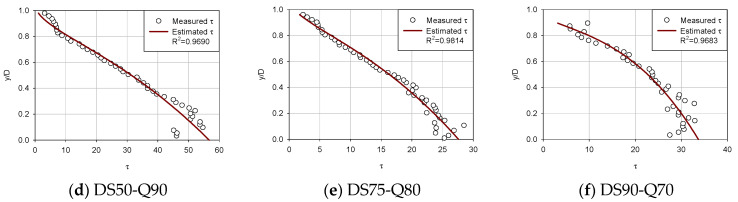
Verification of the proposed shear-stress distribution model with six decelerating non-uniform flows.

**Figure 6:**
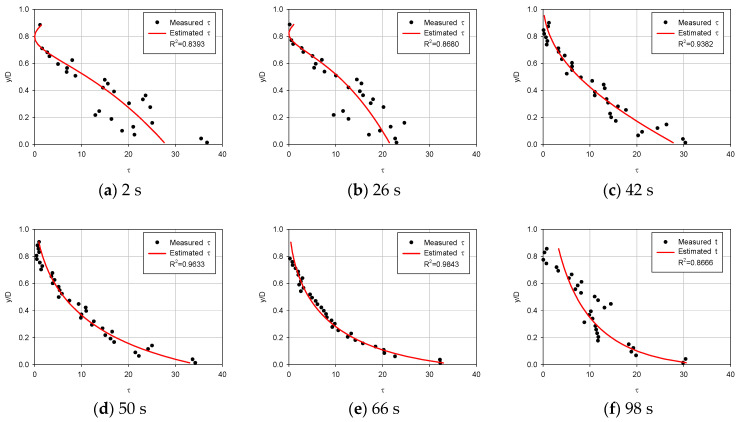
Verification of the proposed shear-stress distribution model with unsteady flow of one slope (S-25-931).

Figure 3, Figure 4, Figure 5 and Figure 6 compare the proposed model with the experimental data of Song [36] to determine if the estimated shear stress distribution fits well with the observed shear stress distribution. Each diagram shows shear stress at the x-axis and y/D at the y-axis with the correlation coefficient of observed data and estimated shear stress.

For first (uniform), second (accelerating non-uniform), third (decelerating non-uniform) and fourth (unsteady) flow conditions (Figure 3, Figure 4, Figure 5 and Figure 6), the proposed model was applied to compare the estimated and observed values of shear stress distribution to see how well they were expressed.

In Figure 3, the proposed shear stress model showed a good agreement with experimental data despite the scattered nature of the data. As for the six sediment runs, the correlation coefficient was actually better in some cases compared to small sediment runs. For uniform flows, the entropy-based model seems to estimate shear stress accurately. In all uniform flows, the correlation coefficient showed a small range from 0.9375 to 0.9931.

From Figure 4, it is found that the proposed model simulated well even in more complex flows. For accelerating non-uniform flows, the entropy-based model seems to estimate shear stress accurately. In all accelerating non-uniform flows, the correlation coefficient showed a small range from 0.9522 to 0.9959. However, some flows have shown very scattered experimental data, especially in the bottom layer due to the difficulties in measuring.

From Figure 5, it can be seen that the entropy-based model simulated very well. For decelerating non-uniform flows, the proposed model seems to compute shear stress accurately. In all decelerating non-uniform flows, the correlation coefficient showed a small range from 0.9475 to 0.9822. The proposed model seems to express very accurate matching results.

From Figure 6, it can be seen that the entropy-based model simulated well. For unsteady flows, the proposed model appears to compute shear stress well. In an unsteady flow (S-25-931), the correlation coefficient was between 0.8262 and 0.9843. The results for the other three slopes are as follows. The correlation coefficient in S-60-933 was between 0.9034 and 0.952, the correlation coefficient in S-10-31 was between 0.8536 and 0.9899, and the correlation coefficient in S-30-932 was between 0.712 and 0.9829. In four cases, the correlation coefficient was above 0.89 on average. This demonstrates the utility of the shear-stress distribution equation using entropy.

### 4.3. Major Parameter Estimation Results

Table 2 shows the result of major parameters from Figure 3, Figure 4, Figure 5 and Figure 6. The entropy-based model showed a 0.9375 to 0.9959 range of correlation coefficients in steady flow conditions and a 0.712 to 0.9899 range of correlation coefficients in unsteady flow conditions. From these results, it seems that the number of measured shear stresses in one distribution does not have a large effect on simulation. Entropy parameter T seems to have a range of −1.441 to 6.405 in steady flow conditions and a range of −5.576 to 4.6124 in unsteady flow conditions. Looking at the correlation coefficients, the decelerating non-uniform flow showed the worst average, 0.9693, whereas accelerating non-uniform flow showed the best average, 0.9887 in steady flow conditions. For unsteady flow conditions, S30-932 showed the worst average, 0.89353, whereas S-10-932 showed the best average, 0.93602.

## 5. Proposal and Verification of Shear Stress Distribution Method

### 5.1. Easily Applied Shear Stress Distribution Formula for Practice

If the main parameters of Table 2 are used in expressions (17) to graph, the distribution of mean shear stress and floor shear stress, ϕ(T), can be obtained based on the slope values of the graph. Using the value of the obtained ϕ(T), it is possible to calculate the river-specific factor ($T^{\prime }$) in Equation (17). Here, $T^{\prime }$ is the average value of T. The entropy parameter T can be obtained from previous equations and represents one cross-section in a river. However, there are various cross-sections in a river which have many values of T, so $T^{\prime }$ is used for a representative factor for one river.

Therefore, in other words, if the river-specific factor $T^{\prime }$ is calculated or known, it is much easier to calculate the boundary shear stress, which is an important hydraulic factor in river design. For example, when the river-specific factor $T^{\prime }$ is used in the shear stress distribution Equations (19) and (20), less calculation and more accurate shear stress distribution can be obtained, because one of the parameters is already known.

**Figure 7:**
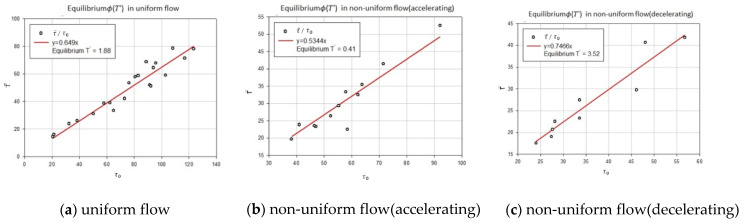
Equilibrium $\emptyset \left(T^{\prime }\right)$ in steady flow.

**Figure 8:**
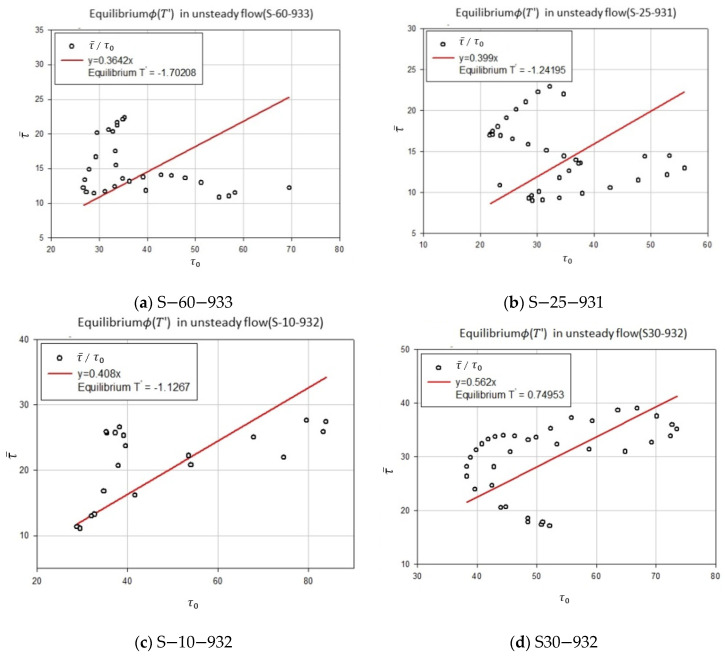
Equilibrium $\emptyset \left(T^{\prime }\right)$ in unsteady flow.

Song data were used to prove the utility of shear stress distribution when the river-specific factor $T^{\prime }$ was fixed. The river-specific factor $T^{\prime }$ for each flow state was then obtained by using the floor shear stress and average shear stress, which are the parameters of Figure 7 and Figure 8. Shear stress was obtained by substituting $T^{\prime }$ for Equations (19) and (20) and the actual measurement value of the Song data was compared and analyzed.

### 5.2. Estimation Graph of River-Specific Factors $T^{\prime }$ in All Flow Conditions

Using Equation (17) based on the data, is the results were displayed graphically to calculate the river-specific factor for each flow state. Figure 7 and Figure 8 show graphs for the calculating of river-specific factors in each flow state.

Figure 7 is a graph of calculating equilibrium $T^{\prime }$ after calculating equilibrium $\phi \left(T^{\prime }\right)$ in uniform, non-uniform accelerating, and non-uniform decelerating flow. For each flow, equilibrium $\phi \left(T^{\prime }\right)$ is 0.6490 (uniform), 0.5344 (non-uniform accelerating), and 0.7466 (non-uniform decelerating); and when Equation (17) is calculated using equilibrium $\phi \left(T^{\prime }\right)$, equilibrium $T^{\prime }$ is calculated as 1.88 (uniform), 0.41 (non-uniform accelerating), and 3.52 (non-uniform decelerating).

Unsteady flow was divided into four cases according to the gradient, and equilibrium $T^{\prime }$ was calculated after selecting equilibrium $\phi \left(T^{\prime }\right)$. Figure 8 is a graph that calculates equilibrium $T^{\prime }$ after obtaining equilibrium $\phi \left(T^{\prime }\right)$ from the unsteady flow. For each case in the unsteady flow, equilibrium $\phi \left(T^{\prime }\right)$ is 0.3642 (S-60-933), 0.399 (S-25-931), 0.408 (S-10-932), and 0.562 (S30-932); and when Equation (17) is calculated using equilibrium $\phi \left(T^{\prime }\right)$, equilibrium $T^{\prime }$ is calculated as −1.70208 (S-60-933), 1.24195 (S-25-931), −1.1267 (S-10-932), and 0.74953 (S30-932).

We now put the equilibrium $T^{\prime }$ values into Equations (19) and (20) and verify the utility of the equilibrium $T^{\prime }$-fixed Equations (19) and (20) through the Song data. If the effectiveness of the equation is verified, the $T^{\prime }$-fixed method easily enables the calculation of the floor shear stress by average shear stress, and the distribution of shear stress can be easily calculated.

### 5.3. Result and Error Analysis

The RMSE is a measure of the residual, which is the difference between the values simulated by the model and actual observed values. The RMSE enables simulative power to be integrated into a single unit of measurement. The RMSE of the model’s simulation for the estimated variable $X_{est,i}$ is defined as the square root of the mean square error Equation (26).

(26) $$\mathrm{RMSE}=\sqrt{\frac{\sum_{i=1}^{n}{\left(X_{obs,i}-X_{est,i}\right)}^{2}}{n}}$$

where $X_{obs,i}$ indicates the actual observed value and $X_{est,i}$ is the simulated value obtained from the model.

The determined shear-stress distribution was compared with those measured by Song. The determined RMSE values were very low from 0.000245 to 0.001108 as shown in Table 3.

Basically, the steady flow shows a high correlation coefficient value and proves the utility of the shear-stress distribution equation. However, the unsteady flow shows a lower mean correlation value compared to the steady flow. Looking at the results of the unsteady flow in detail, note that not all areas are observed with low correlation, but with high and low correlation. This indicates that the low correlation coefficient value from the unsteady flow is the error caused by the observation of the data. In practice, using point shear stress values calculated based on accurate observations can reduce error and have higher correlation coefficient values.

Based on the advantages of being able to express and easily obtain shear stress distribution by entropy shear stress distribution using equilibrium $T^{\prime }$, it is suggested that the contents of tractive force and critical tractive force with unclear accuracy can be revised to shear stress in Korea’s river design standards.

## 6. Discussion

The proposed equation has proved to show reasonable results. From Figure 3, Figure 4, Figure 5 and Figure 6, in most cases, there is a good match between Song’s observed data and shear stress values from the proposed model. It was confirmed that shear stress distributions estimated are accurate showing an average 0.9780 correlation coefficient for forty-two types of steady open channel flows. In four cases where the flow was unsteady, the mean value of the correlation coefficient was found to be 0.9084. The estimated values are not from an empirical formula but from a theoretical method, which has a great meaning for open channel fields.

Using the estimated boundary shear stress and mean shear stress from all forty-two runs, entropy parameter T was analyzed as 1.629. Figure 7 and Figure 8 were plotted using the relationship between bed shear stress and mean shear stress, Equation (17), which seems to have a tendency for an equilibrium state. The equilibrium state of a velocity distribution was studied previously by Chiu et al. [38,39], which shows similar results.

The aforementioned discussion delineates that an entropy-based model on shear stress is able to describe the characteristics of shear stress from the frictional force caused by viscosity and fluctuating fluid velocity in an open channel turbulent flow. The proposed equation has proved to show reasonable results.

Based on the parameters calculated, the mean values of the correlation coefficient in the results of the shear stress distribution equation for each flow were 0.9708 for the uniform flow, 0.9867 for the non-uniform flow (accelerating), 0.9471 for the non-uniform flow(decelerating), and 0.8604–0.9212 in the unsteady flow for each gradient. Based on these results, it was confirmed that the shear stress distribution formula using river-specific factors can be available in practice.

Of course, part of the unsteady flow shows lower correlation coefficient values, but the correlation coefficient values are error generated from observations. When measuring the distribution of point shear stress for practical use, the conclusions based on accurate observations are determined to reduce the error further and obtain high accuracy shear stress distributions and results.

However, there are some minor limitations to this study. Some observed data, such as shear stress distribution and vertical depth from the bed, should be known in order to estimate the model. In other words, there needs to be some information in order to use this model. In addition, the basic shape of the model depends on the PDF of shear stress and Equation (21). This is important because in complex flows such as unsteady flows, it can be difficult to show the distribution exactly, especially if the maximum velocity occurs below the water surface. The model is based on probability and statistics and does not consider basic hydraulics in the beginning, but only depends on constraints and data. Because the accuracy of the observed data can affect the correlation value of the distribution equation, accurate point shear stress data must be obtained for use in practice.

## 7. Conclusions and Proposition

Although the tractive force and critical tractive force formulas used in the current Korean river design standards are simple to obtain, the tractive force is a formula using a factor that is difficult to obtain, which makes it difficult to calculate the exact value. In addition, critical tractive force is a value obtained empirically, which also has the disadvantage of difficulty in calculating the exact value. However, in order to use the existing shear stress formula, shear stress can be calculated only with the energy gradient, a factor that is very difficult to obtain, so Korea’s river design standards use tractive force and critical tractive force instead of shear stress.

Therefore, using the entropy concept, this study has proposed shear stress distribution and boundary layer shear stress, which can be applied in open channel flows. To determine how well the shear stress model fits with the observed data, Song’s data were used. From this aspect, this paper validated the model with a wide range of forty-two runs of experimental data published in the literature. The results show the utility and reliability of Equations (19) and (20), in which the mean shear stress is considered, then using the qualified shear stress distribution, the shear stress distribution over the whole flow depth of open channel turbulent flows is shown.

Furthermore, based on the equations’ proven utility and reliability, this paper has proposed a method that can easily be used in practice by obtaining river-specific factor T if point shear stress is given. We suggest that the contents of tractive force and critical tractive forces with shortcomings in terms of accuracy in the Korean river design standards are revised to the shear stress distribution method presented in this study. The results of the distribution of shear stress after the calculation of river-specific factor T were also considered to be highly correlated, and thus it was determined that the method could be used in practice.

However, there is no best entropy parameter T value for every run. This means that we need to somewhat reach an agreement with solutions. To improve this, very precise methods must be applied in order to enhance the results. One of these methods can be an optimum technique. Optimization techniques such as genetic algorithms or harmony searching can be applied for finding better solutions to these kinds of problems, where we might even be getting closer to what we are seeking.

There have been very few studies about shear stress in open channels recently. It seems that there are only few models that can estimate shear stress distribution. Some of the models require parameters that are difficult to calculate or even obtain, or even have hypotheses that eventually reach the limit of the formula. Other models have many parameters to estimate, but the features of the proposed model have only two parameters, the entropy parameter (*T*) and the unknown boundary shear stress or mean shear stress.

In addition, if the river-specific factors T are calculated for practical use based on the proposed model, the boundary shear stress, which is an important river factor for river design, can be calculated immediately, and shear stress distribution can easily be calculated. The proposed model can be used regardless of the shape or flow of the river (except for unsteady flow). Nevertheless, in future research, our model has to be compared with some shear stress models.

The analysis has limitations but the results appear to be useful. If the point shear stress in the open channel and vertical depth from the bed are given, the shear stress distribution can be estimated simply from the model, which will show high availability when designing or managing the open channel. In addition, the boundary shear stress can be estimated easily without the energy gradient when calculating the boundary shear stress in an open channel.

## Figures and Tables

**Table 1:** Experimental data.

Flow Type		Slope (%)	Discharge (cm/s)	Sediment (cm)
Uniform	S25-Q40	0.25	40	12.2
	S50-Q50	0.5	50	11.8
	S75-Q120	0.75	120	18.5
	S90-Q70	0.90	70	12.2
	S100-Q55	1.00	55	10.5
	S125-Q50	1.25	50	9.1
Non-uniform (accelerating)	AS00-Q80	0	80	14.5
	AS-25-Q100	0.25	100	16.9
	AS-25-Q80	0.25	80	15.1
	AS-50-Q110	0.50	110	17.9
	AS-75-Q80	0.75	80	16.5
	AS-93-Q80	0.93	80	17.2
Non-uniform (deaccelerating)	DS25-Q70	0.25	70	16.0
	DS25-Q90	0.25	90	20.0
	DS50-Q70	0.50	70	16.5
	DS50-Q90	0.50	90	18.5
	DS75-Q80	0.75	80	20.5
	DS90-Q70	0.90	70	18.0
Unsteady	S-25-931	0.631–0.697	4.05	6.26–7.04
	S-60-933	0.564–0.612	7.01	5.11–6.14
	S-10-932	0.642–0.703	5.74	5.94–7.63
	S30-932	0.821–0.944	5.70	6.34–7.66

**Table 2:** Summary of major parameter results for steady (right) and unsteady (left) flow conditions.

Data Set	$\tau_{0}$	$\bar{\tau }$	T	$R^{2}$	Data Set	$\tau_{0}$	$\bar{\tau }$	T	$R^{2}$
S-60-933(t1)	69.5773	12.2146	−5.5760	0.9491	S25-Q31	20.531	14.458	2.742	0.9375
S-60-933(t3)	58.2623	11.4943	−4.8793	0.9340	S25-Q40	21.519	16.134	3.588	0.9834
⋮	⋮	⋮	⋮	⋮	⋮	⋮	⋮	⋮	⋮
S-60-933(t57)	47.9180	13.5993	−2.9491	0.9512	S150-Q40	102.850	58.927	0.887	0.9809
S-60-933(t59)	51.2248	12.9439	−3.5383	0.9520	S150-Q50	123.535	78.079	1.656	0.9677
S-10-932(t2)	39.5775	23.7430	1.2288	0.9255	AS00-Q145	92.144	52.484	0.845	0.9957
S-10-932(t6)	39.2120	25.3055	1.8404	0.9176	AS00-Q100	71.481	41.464	0.976	0.9934
⋮	⋮	⋮	⋮	⋮	⋮	⋮	⋮	⋮	⋮
S-10-932(t54)	41.6155	16.2094	−1.3667	0.9867	AS-93-Q100	41.036	23.846	0.989	0.9829
S-10-932(t78)	37.9574	20.7072	0.5492	0.9267	AS-93-Q80	38.194	19.674	0.182	0.9902
S-25-931(t2)	28.0111	21.0477	3.6227	0.8393	DS25-Q90	27.408	19.021	2.573	0.9769
S-25-931(t58)	37.6556	13.5880	−1.75	0.9800	DS25-Q70	23.983	17.553	3.236	0.9822
⋮	⋮	⋮	⋮	⋮	⋮	⋮	⋮	⋮	⋮
S-25-931(t134)	32.2337	22.9028	2.8501	0.8262	DS90-Q80	48.087	40.659	6.405	0.9734
S-25-931(t138)	30.1916	22.2638	3.3415	0.8382	DS90-Q70	33.711	27.751	5.532	0.9683
S-30-932(t1)	39.6605	23.9780	1.2893	0.9098					
S-30-932(t17)	44.3978	33.9348	3.9019	0.7628					
⋮	⋮	⋮	⋮	⋮					
S-30-932(t57)	44.8305	20.6737	−0.4679	0.9607					
S-30-932(t59)	48.5676	17.8320	−1.6665	0.9461					

**Table 3:** Simulation results R^2^ and RMSE.

Flow Type		R^2^	RMSE
Uniform	S25-Q40	0.9834	0.000491
	S50-Q50	0.9848	0.000431
	S75-Q120	0.9908	0.000394
	S90-Q70	0.9812	0.000424
	S100-Q55	0.9721	0.000564
	S125-Q50	0.9871	0.000417
Non-uniform (accelerating)	AS00-Q80	0.9959	0.000284
	AS-25-Q100	0.9933	0.000304
	AS-25-Q80	0.9914	0.000346
	AS-50-Q110	0.9957	0.000245
	AS-75-Q80	0.9931	0.000364
	AS-93-Q80	0.9902	0.000384
Non-uniform (deaccelerating)	DS25-Q70	0.9822	0.000436
	DS25-Q90	0.9769	0.000513
	DS50-Q70	0.9670	0.000685
	DS50-Q90	0.9690	0.000613
	DS75-Q80	0.9814	0.000531
	DS90-Q70	0.9683	0.000678
Unsteady	S-25-931 2 s	0.8393	0.001108
	S-25-931 26 s	0.8680	0.001085
	S-25-931 42 s	0.9382	0.000712
	S-25-931 50 s	0.9633	0.000675
	S-25-931 66 s	0.9643	0.000645
	S-25-931 98 s	0.8666	0.000907

## Data Availability

Not applicable.
